# Biological reaction control using topography regulation of nanostructured titanium

**DOI:** 10.1038/s41598-020-59395-4

**Published:** 2020-02-12

**Authors:** Mayuko Shiozawa, Haruka Takeuchi, Yosuke Akiba, Kaori Eguchi, Nami Akiba, Yujin Aoyagi, Masako Nagasawa, Hiroyuki Kuwae, Kenji Izumi, Katsumi Uoshima, Jun Mizuno

**Affiliations:** 1 Faculty of Science and Engineering, Waseda University, 3-4-1 Okubo, Shinjuku, Tokyo, 169–8555 Japan; 2 Division of Bio-prosthodontics, Department of Oral Health Science, Niigata University Graduate School of Medical and Dental Sciences, 2-5274, Gakkocho-dori, Chuo-ku, Niigata, 951-8514 Japan; 3 Division of Biomimetics, Graduate School of Medical and Dental Sciences, Niigata University, 2-5274, Gakkocho-dori, Chuo-ku, Niigata, 951-8514 Japan; 4 Research Organization for Nano & Life Innovation, Waseda University, 513, Waseda-tsurumaki-cho, Shinjuku-ku, Tokyo, 162-0041 Japan

**Keywords:** Implants, Collective cell migration

## Abstract

The micro- and nanosize surface topography of dental implants has been shown to affect the growth of surrounding cells. In this study, standardized and controlled periodic nanopatterns were fabricated with nanosized surface roughness on titanium substrates, and their influence on bone marrow stromal cells investigated. Cell proliferation assays revealed that the bare substrate with a 1.7 nm surface roughness has lower hydrophilicity but higher proliferation ability than that with a 0.6 nm surface roughness. Further, with the latter substrate, directional cell growth was observed for line and groove patterns with a width of 100 nm and a height of 50 or 100 nm, but not for those with a height of 10 or 25 nm. With the smooth substrate, time-lapse microscopic analyses showed that more than 80% of the bone marrow cells on the line and groove pattern with a height of 100 nm grew and divided along the lines. As the nanosized grain structure controls the cell proliferation rate and the nanosized line and groove structure (50–100 nm) controls cell migration, division, and growth orientation, these standardized nanosized titanium structures can be used to elucidate the mechanisms by which surface topography regulates tissue responses to biomaterials.

## Introduction

Since osseointegration was first observed in 1952^1^, pure titanium has been used as a biomaterial, especially for dental implants in the dental field. Titanium has a high resistance to corrosion and is widely regarded as the most biocompatible metal because a stable and inert oxide layer spontaneously forms when its surface is exposed to an oxidizing medium^2^. Dental implants are an effective treatment strategy for functional and aesthetic recovery. Achieving osseointegration is the first essential step of successful dental implant therapy. To accelerate osseointegration, companies and researchers have tried to improve the surface topography of the implant body. Microroughness is introduced onto commercially distributed implants by blasting and/or etching with the expectation of improved cell adhesion and bone formation. Several *in vitro* and *in vivo* animal studies have shown that titanium surfaces with nanoscale topographies promote cell adhesion^3^, osteogenic differentiation^4^, and bone formation^5^. If the mechanisms regulating the surrounding cells and tissues by nanoscale surface structures are elucidated, the biological reactions of titanium-attached cells can be controlled by an effective nanostructure surface design.

However, owing to technical limitations, conventional surface topographies have been found to consist of non-standardized and random structures. Therefore, the detailed mechanisms by which nanoscale structures promote bone formation are not well understood. To understand these mechanisms and achieve intentional control of cell activity by using the surface structure of the implant, standardized, controlled, and periodic nanosized titanium structures should be fabricated. Several studies^6^ on the fabrication of nanosized patterned titanium structures have been reported, but these structures were relatively rough and larger than 100 nm. In this study, smaller, standardized, controlled, and periodic nanosized structures on titanium surfaces were successfully fabricated. Subsequently, the effects of fine, organized, standardized, and controlled nanosized titanium structures on cell growth were analyzed. Further, live imaging of cell alignment, growth, and activity on these substrates was performed. This study provides a basis for the design of effective nanostructures for optimal osseointegration of dental implants.

## Results

### Scanning electron microscopy images of substrates

**Figure 1:**
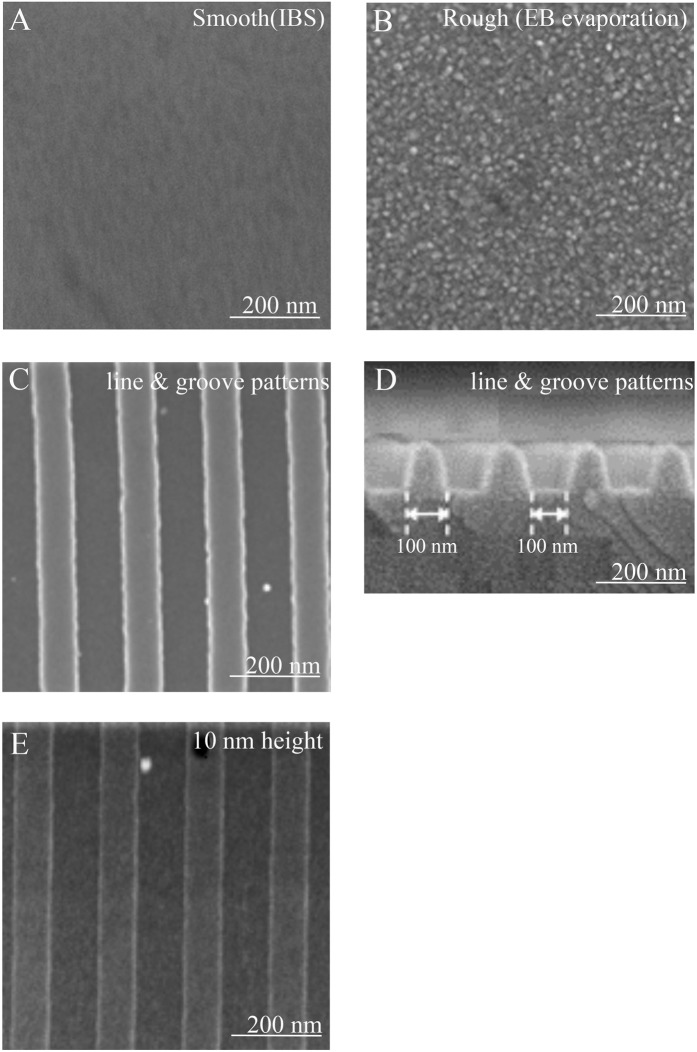
Scanning electron microscopy images of titanium substrates. Bare flat surfaces of titanium substrates with (**A**) 0.6 nm surface roughness with no grains and (**B**) 1.7 nm surface roughness with single nanosized grains. Titanium substrates with a line and groove pattern with a height of 100 nm (**C**) and 200 nm pitch (**D**), and line and groove pattern with a height of 10 nm (**E**).

Both ion beam sputtering (IBS) and electron beam (EB) evaporation result in titanium substrates with very smooth surfaces, as revealed by low-magnification scanning electron microscopy (SEM) images. However, there is a small difference in the surface roughness of the substrates, namely, 0.6 nm surface roughness with IBS (Figs. 1A) and 1.7 nm surface roughness with EB evaporation (Fig. 1B). In the 0.6 nm surface roughness substrate, no grains could be seen on the substrate (Fig. 1A). In contrast, small grains (~10 nm) were observed on the 1.7 nm surface roughness substrate (Fig. 1B). Line and groove patterns were fabricated on the titanium substrates with IBS (0.6 nm surface roughness). The structures had a width of 100 nm, a pitch of 200 nm, and a height of 100 (Fig. 1C,D), 50, 25, or 10 nm. Even the line and groove pattern with a height of 10 nm exhibited a clear stripe pattern (Fig. 1E).

### Hydrophilic properties of substrate

**Figure 2:**
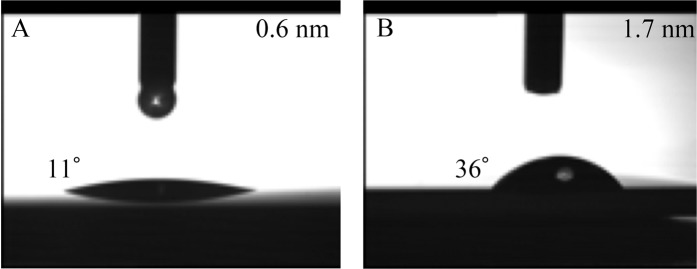
Water contact angles on bare titanium substrates. Substrate with surface roughness (**A**) of 0.6 nm and (**B**) of 1.7 nm.

The hydrophilicity of a sample may be determined from the contact angle of water on the surface. On a superhydrophilic surface, water will spread completely across the surface rather than forming droplets. The titanium substrate with a surface roughness of 0.6 nm exhibited a very low water contact angle (<11°) (Fig. 2A), whereas higher water contact angles (<36°) were observed for the titanium substrate with a surface roughness of 1.7 nm (Fig. 2B). This result indicates that both titanium surfaces were hydrophilic, although the substrate with the 0.6 nm surface roughness could exhibit superhydrophilic capabilities.

### Cell proliferation analysis

**Figure 3:**
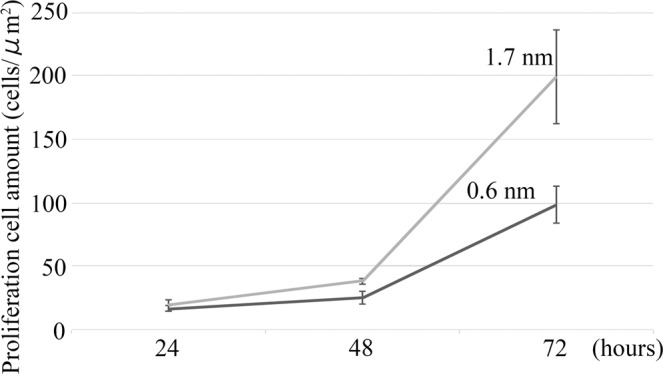
Cell proliferation analysis. The 1.7 nm surface roughness substrate shows a higher cell proliferation ability than the 0.6 nm surface roughness substrate.

The cell viability on the flat titanium substrates was determined by cell proliferation analyses (Fig. 3). After 24 hours, the smooth (0.6 nm surface roughness) substrate and rough (1.7 nm surface roughness) substrate exhibited similar cell proliferation rates (16.42 ± 2.11 vs 19.14 ± 4.55 cells/µm^2^). After 48 hours, a slightly higher number of cells was detected on the rough substrate (25.73 ± 5.51 vs 38.28 ± 2.14 cells/µm^2^) (P :0.0069 < 0.05). After 72 hours, the bone marrow stromal cell (BMSC) exhibited a much higher proliferation ability on the rough surface (98.52 ± 13.73 vs 199.37 ± 32.12 cells/µm^2^) (P:0.0002 < 0.05).

### Cell alignment ability of the nanoscale line and groove patterned titanium substrates

**Figure 4:**
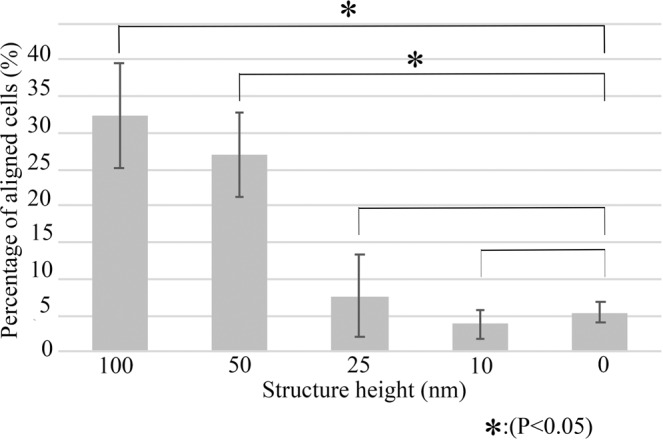
Cell alignment control ability of line and groove patterns on titanium substrates.

BMSCs were cultured on the smooth (0.6 nm surface roughness) titanium substrates with line and groove patterns (width: 100 nm, pitch: 200 nm, and height: 10, 25, 50, or 100 nm). A flat substrate was used as the control. BMSCs were seeded on the titanium substrate at a concentration of 1.0 × 10^4^ cells/µL and cultured for 72 hours. The cells were fixed and then observed under a light field microscope. On the substrates with heights of 50 and 100 nm, 34% of the BMSCs were oriented along the line and groove pattern. These amounts were significantly different from the number of cells aligned on the flat substrate (100 nm vs 0 nm: 32.21 ± 7.21 vs 5.36 ± 1.38: P:0.0012 < 0.05; 50 nm vs 0 nm: 27.01 ± 5.77 vs 5.36 ± 1.38: P:0.0023 < 0.05). By contrast, no significant differences were observed between the oriented cells detected on the patterns with heights of 10 and 25 nm and the flat substrate (25 nm vs 0 nm: 7.60 ± 5.62 vs 5.36 ± 1.38; 10 nm vs 0 nm: 3.77 ± 1.8 vs 5.36 ± 1.38) (Fig. 4).

### Immunocytochemistry

**Figure 5:**
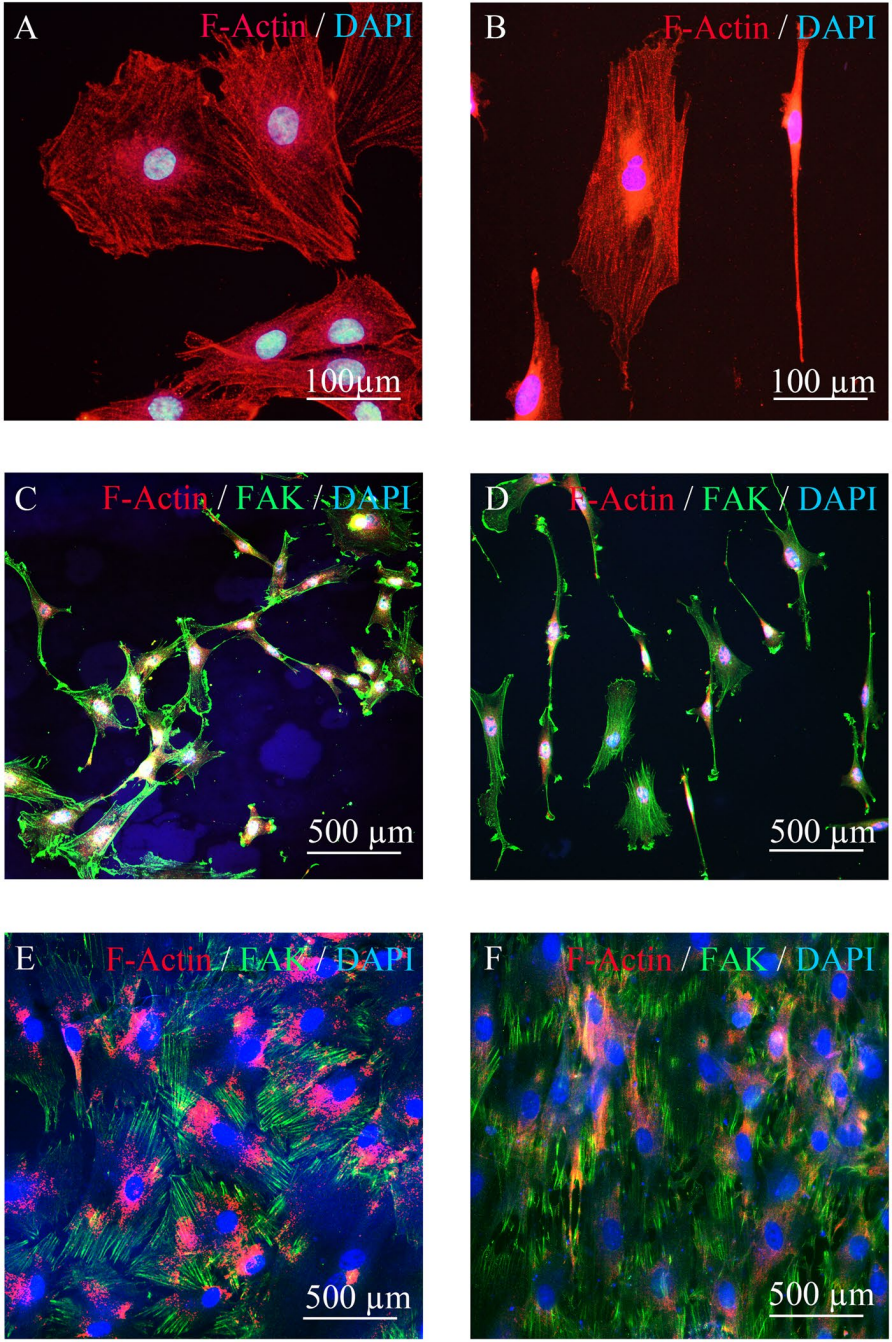
Histological analysis with fluorescence immunocytochemistry. The BMSCs were cultured on a flat substrate (**A,C,E**) or a substrate with a line and groove pattern (**B,D,F**).

Twenty-four or 48 hours after seeding the BMSCs on the line and groove pattern with a height of 100 nm or a flat pattern on the smooth (0.6 nm surface roughness) substrate, the cells were fixed and stained with anti-focal adhesion kinase (FAK) and/or anti-actin filament (F-Actin) antibodies. At the low concentration conditions after 24 hours, some BMSCs on the flat substrate had extended and F-actin filaments in the BMSCs ran in multiple directions (Fig. 5A). FAK was mainly detected along the edge in multiple directions (Fig. 5C). At the confluent conditions after 48 hours, multidirectional fibrous structures from the edge to the nuclei were detected (Fig. 5E). Further, 24 hours after seeding, the BMSCs on the 100 nm high line and groove pattern on the rough substrate showed spindle and extended shapes (Fig. 5B) with F-actin filaments aligned along the line and groove pattern (Fig. 5B). Under both low and high concentration conditions, FAK at the edge of the cells, which has a fibrous structure, was observed to align with the line and groove pattern (Figs. 5D,F).

### Live imaging analysis

**Figure 6:**
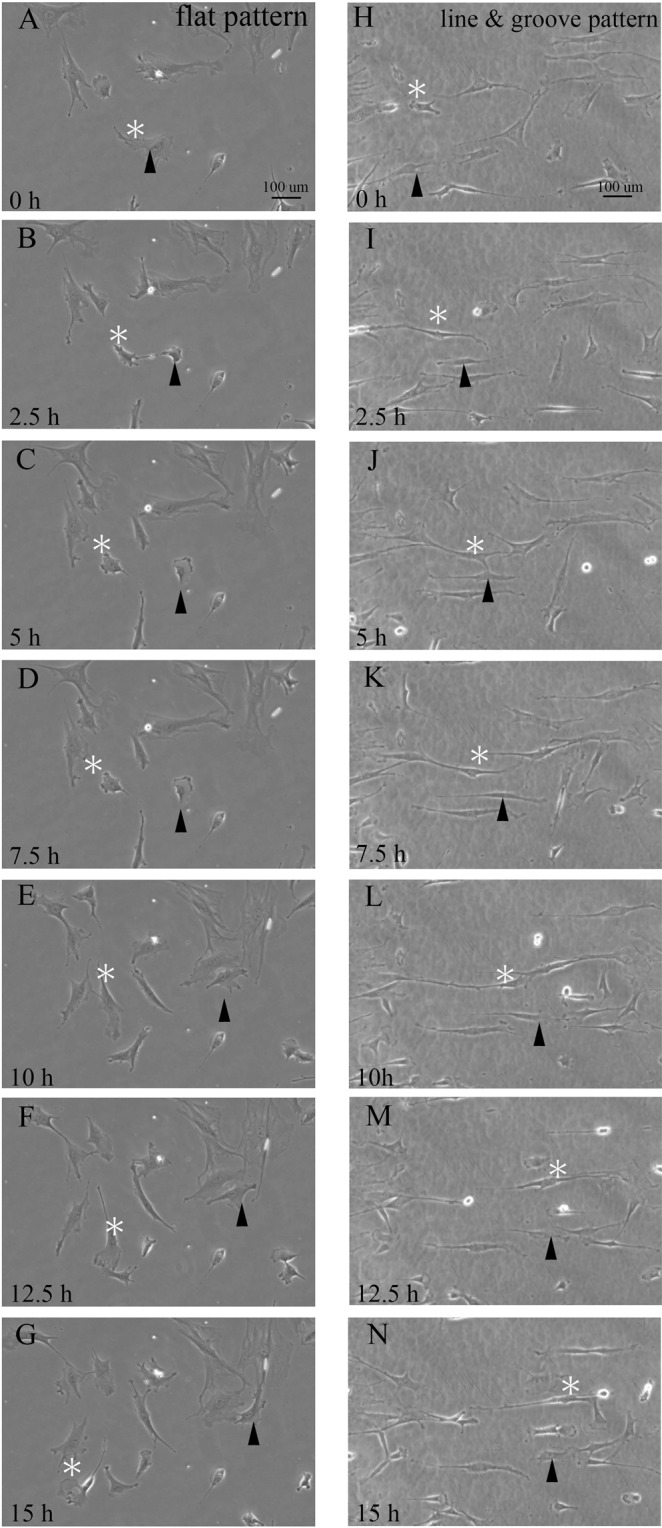
Time-lapse images of migrating BMSCs cultured on titanium substrates for 15 hours. Serial images of migrating and proliferating BMSCs cultured on a flat bare substrate (**A–G**) (Time laps image of 72 hours culture. Movie S1: supplemental) or a line and groove patterned substrate (**H–N**) (Time laps image of 72 hours culture. Movie S2: supplemental).

To examine the cell growth ability of the substrates with nanosized titanium surface structures, live imaging analyses were performed. The BMSCs were seeded on the 100 nm high line and groove pattern or a flat pattern on the 0.6 nm surface roughness substrate. During the 72 hours culture period, the BMSCs on the flat pattern titanium substrate migrated, grew, and divided in random directions, as shown in Movie S1 (supplementary). Figures 6A–G indicate 15 hours cell growth on flat pattern titanium substrate. BMSCs migrated and grew in different directions, as indicated by the white asterisks and black arrow heads (Fig. 6A–G). By contrast, almost all the cells on the 100 nm high line and groove pattern titanium substrate showed directional migration, growth, and oriented cell division along the line and groove pattern until confluence conditions were reached, as shown by the white asterisks and black arrow heads in Fig. 6H–N and Movie S2 (supplementary). Even after confluency, directional and orientational cell activities could be observed, but not clearly. Interestingly, spindle-shaped cells always migrated following the patterns, whereas some flat-shaped cells could move across the patterns.

## Discussion

Titanium is commonly used for dental implants and the nanoscale surface topography of titanium dental implants may affect the activity of the attached cells. Previous studies have demonstrated a relationship between nanosized topography and accelerated osteogenic differentiation^7,8^. However, the details of the mechanisms of this relationship remain unknown; this is because of the irregularity of the nanostructures. Therefore, a technology that can fabricate standardized accurate and repeating structures is necessitated, which will facilitate the elucidation of these mechanisms. There have been numerous reports that have attempted to fabricate standardized nanostructures^6,9,10^. However, instead of pure titanium, a titanium alloy or other metal alloy was used^9^. Even when titanium was used, the sizes of the nanostructure in these studies were larger than 100 nm or rough structures were fabricated^6,10^. In this study, we fabricated fine, accurate, and smaller structures on a grade 4 pure titanium substrate using UV-nanoimprint lithography (UV-NIL), reactive ion etching (RIE), IBS, and EB evaporation. This approach allowed control of the surface roughness (formation of a single size of nano grains) and control of the structure.

We used the treated substrates to examine the effects of such accurate nanoscale structures on cell growth. First, we determined BMSCs proliferation by comparing bare titanium substrates with smooth (0.6 nm surface roughness) and relatively rough (1.7 nm surface roughness) surfaces. In the context of this study, the 1.7 nm surface roughness substrate was defined as “rough.” However, the plates employed in previous studies exhibited a significantly coarser surface and a height difference of up to 10 nm was observed for a bare plate of 1.7 nm surface roughness; these plates were polished mechanically^7–9^. The 0.6 nm surface roughness substrate exhibited higher hydrophilicity than the 1.7 nm surface roughness substrate. Although previous studies have reported that high hydrophilicity aids cell proliferation^11,12^, our results showed that the 1.7 nm surface roughness substrate that has lower hydrophilicity promoted higher cell proliferation than the 0.6 nm surface roughness substrate that has no grains. Undoubtedly, wettability is affected not only by surface roughness but also by the nanostructure. Further research will help us to clarify the relationships between surface roughness, nanostructure, and cell proliferation. Conceivably, a 0.6 nm surface roughness on bare plates with no grains can be considered extremely smooth for cell adhesion, proliferation, and growth. On the other hand, a super smooth surface without any grains might aid hydrophilicity. Even 1.7 nm surface roughness bare plate showed lower hydrophilicity, nano size grains might help cell proliferation. For our purpose, we wanted to observe cell growth on nanosized titanium structures in extreme conditions. Therefore, we applied a 100 nm line and groove structure on the 0.6 nm surface roughness substrate and used the bare titanium plate as the control.

BMSCs were seeded on four titanium substrates with line and groove patterns of decreasing groove heights (100, 50, 25, and 10 nm), to determine the threshold at which the BMSCs perceive the nanoscale patterns. Approximately 30% of the BMSCs were oriented along the 100 and 50 nm high line and groove structures on the 0.6 nm grain size substrate. Our results reveal that the structure height should be more than 25 nm for orientational control by the structure on the titanium substrate. The immunocytochemistry analyses also showed alignment of the cytoskeletal factors F-actin and FAK in the BMSCs, which sense nanosized patterns through adhesion molecules such as integrin until confluent conditions. The sense organs and/or sensitivities for cell proliferation and cell growth orientation are expected to differ. Live cell imaging analyses showed that more than 80% of the cells were directed by the 100 nm high line and groove structure on the smooth substrate (0.6 nm surface roughness). Surprisingly, the migration and division of spindle-shaped and oriented cells were also directed along the line and groove pattern until over the confluent conditions. Even over the confluent conditions, the BMSCs appeared to move along the patterns for at least 72 hours. However, some flat-shaped cells could move across the pattern direction. Matsugaki *et al*. showed that collagen fibers orient orthogonal to the osteoblast alignment on Ti-6Al-4V nanogrooved structures^10^. Other previous studies have shown that extracellular matrix (ECM) organization follows the cell direction^13,14^. These results suggest the possibility that the oriented cells that were directed and migrated along the nanogroove patterns were different from ECM secreted cells. Additional work is required to elucidate the relationship between cell growth and ECM secretion.

In this study, standardized and precise nanosized structures were successfully fabricated on titanium. Moreover, cell growth and migration control on nanosized line and groove structures was observed. However, to elucidate the relationship between nano-surface roughness or structures and cell proliferation as well as growth control, various grain sizes and nanostructures (size, pattern, and pitch) should be examined. It is considered that depending on the cell source and differentiation stage, the sensitivity and preference against surface roughness and/or nanostructure would differ. Using our technology, we can fabricate several degrees of surface roughness and structures at the nanoscale. Further studies are required to elucidate these relationships.

As a starting point, our study indicated that subnanosize surface roughness controlled cell proliferation and 50 nm size structures could control cell growth orientation. These *in vitro* results will provide insight into the mechanisms by which tissue formations are controlled by nanosized structures on dental implants and promote the development of novel functional biomaterials.

## Conclusions

In this study, a threshold height for the nanostructures for cell alignment was determined. Cell migration and division were observed along the nanosized line and groove pattern. The proliferation of BMSCs was promoted by nanosized surface roughness and cell orientation was affected by structures that were more than 50 nm high. Further, the nanosized line and groove pattern controlled not only cell alignment but also the migration and division direction. These findings provide new insights into the use of patterning technology for the control of cell responses to biomaterials.

## Materials and Methods

### Titanium substrates

Grade 4 pure titanium substrates were prepared at the Research Organization for Nano & Life Innovation, Waseda University. Nanosized patterns were fabricated by UV-NIL and RIE according to our previous report^15^. First, a 200 nm thick silicon dioxide layer was deposited by IBS on a glass substrate as a nano structuring layer. An etching mask for the nanosized pattern was formed by UV-NIL using a UV curable resin. Then, the etching mask pattern was transferred to the silicon dioxide layer via RIE using a mixture of octafluoropropane gas and oxygen gas. Subsequently, the remaining etching mask was removed using oxygen gas. By applying these techniques, we were able to fabricate repetitive nanoscale structures such as lines and grooves on the substrate. IBS for high energy deposition or EB evaporation for low energy deposition was used to finish the grade 4 pure titanium surface^16^. The metal grain size depended on the energy of the impinging metal atoms^17^. IBS fabricated a smooth surface with a surface roughness of 0.6 nm, whereas EB evaporation fabricated a rough surface (~10 nm particles) with a surface roughness of 1.7 nm. In this study, we prepared substrates with flat patterns or line and groove patterns with a width of 100 nm, pitch of 200 nm, and height of 10, 25, 50, or 100 nm on the smooth (0.6 nm surface roughness) substrate. The topologies of the prepared titanium surfaces were examined using SEM (Hitachi High-Technologies, Japan). The contact angles of the surfaces were characterized using a contact angle meter (Kyowa Interface Science, Japan) with 2 µL of deionized water at room temperature.

### Cell cultures

BMSCs were collected from the femoral bone of a 4 week-old male SD rat (Charles River, USA). The BMSCs were flushed out from the bone and cultured with cell proliferation medium (alpha-MEM (Gibco) containing 10% heat-inactivated fetal bovine serum (Gibco) and 1% antibiotic antimycotic solution (Sigma)) at 37 °C in a 5% CO_2_ humidified incubator. The BMSCs were allowed to adhere to a plastic support for 24 hours. Nonadherent cells were removed by flushing with alpha-MEM and the medium was replaced every 3 days. The cells were passaged and used for experiments within passage 3. The cells were seeded on the nanopatterned titanium substrates at a concentration of 1 × 10^4^ cells/ml and then cultured for 72 hours in cell proliferation medium.

### Cell proliferation analysis

Images of the cells were collected 24, 48, and 72 hours after seeding using a light field microscope, and the cell numbers and concentrations were analyzed using the ImageJ software (NIH, USA)^18^. The images were also used to analyze the orientation of cell growth. The cell growth orientation was defined relative to the long axis of the spindle-shaped BMSCs. Cells aligned along the line and groove pattern within ± 10° were identified as oriented cells.

### Immunocytochemistry

Cells cultured on the titanium substrate were fixed with 10% formaldehyde. The activation of FAK and F-Actin was analyzed by immunocytochemistry. FAK and F-actin in the BMSCs were stained with mouse anti-FAK monoclonal antibodies (1:1000) (Santa Cruz, Boston, USA) or rabbit anti-F-actin polyclonal antibodies (1:1000) (Bioss Antibodies, Woburn, USA) as primary antibodies and Alexa Fluor 488 or 594 (1:500) (Bioss Antibodies, Woburn, USA) as secondary antibodies. Nucleolus were stained with DAPI (Abcom, Cambridge, UK).

### Real-time imaging of cell growth

Real‐time imaging of the BMSCs cultured on the titanium substrate in 6‐well plates was performed using a Keyence BZ‐X700 all‐in‐one fluorescence microscope equipped with a CO_2_‐ and temperature‐controlled chamber and a time‐lapse tracking system (Keyence, Osaka, Japan). Phase contrast images were taken every 15 min for 72 hours and converted to movie files using a BZ‐X Analyzer (Keyence).

### Statistics

Data are expressed as means ± standard deviation (SD). Student’s t-tests were performed to analyze the differences between two groups. Multiple-group comparisons were performed using one-way analysis of variance (ANOVA). P values of less than 0.05 were considered to be significant.

### Ethical approval

This study was approved by the President of Niigata University after a review by the Institutional Animal Care and Use Committee (Permission number:SA00366), and was carried out according to the Niigata University Animal Experimentation Regulations.

## Supplementary information

Supplementary Videos (Movie S1).

Supplementary Videos (Movie S2).
